# Noninvasive prenatal testing, ultrasonographic findings and poor prenatal diagnosis rates for twin pregnancies: a retrospective study

**DOI:** 10.1186/s12884-023-05642-1

**Published:** 2023-05-13

**Authors:** Xiying Yuan, Weinan Wang, Lei Dai, Wenjing Yong, Chenlin Pei, Jingzhi Li, Lingqian Wu

**Affiliations:** 1 Department of Obstetrics, Xiangya Hospital, Central South University, Changsha, Hunan China; 2 Center for Medical Genetics and Hunan Key Laboratory of Medical Genetics, School of Life Sciences, Central South University, Changsha, Hunan China; 3 Department of Medical Genetics, Hunan Jiahui Genetics Hospital, Changsha, Hunan China

**Keywords:** Noninvasive prenatal testing, Ultrasonographic findings, Prenatal diagnosis rate, Twin pregnancies

## Abstract

**Background:**

Noninvasive prenatal testing (NIPT) is increasingly used in the clinical prenatal screening of twin pregnancies, and its screening performance for chromosomal abnormalities requires further evaluation. For twin pregnancies with indications for prenatal diagnosis, there is a lack of clinical data to assess the prenatal diagnosis rate (PDR). The aim of this study was to evaluate the screening performance of NIPT for foetal chromosomal abnormalities in twin pregnancies and the PDR in the second and third trimesters.

**Methods:**

Ultrasound scans were carried out for all twin pregnancies between 11 and 13^+ 6^ gestational weeks. For twin pregnancies with nuchal translucency thickness˂3.0 mm and no foetal structural malformations, NIPT was performed after blood sampling, followed by routine ultrasound monitoring. Women with twin pregnancies who underwent NIPT at the prenatal diagnostic centre of Xiangya Hospital from January 2018 to May 2022 were included in the study. Genetic counselling was offered to each pregnant woman when the NIPT result indicated a high risk of abnormalities or abnormal ultrasonographic (USG) findings were detected. We followed up twin pregnancies for NIPT results, USG findings, prenatal diagnosis results and pregnancy outcomes.

**Results:**

In 1754 twin pregnancies, the sensitivity, specificity and positive predictive value of NIPT for trisomy 21 were 100%, 99.9% and 75%, and the corresponding values for sex chromosome aneuploidy (SCA) were 100%, 99.9% and 50%, respectively. For the 14 twin pregnancies for which the NIPT results indicated a high risk of abnormalities, the PDR was 78.6% (11/14). For the 492 twin pregnancies for which the NIPT results indicated a low risk of abnormalities, the rate of USG findings in the second and third trimesters was 39.4% (194/492); of these pregnancies, prenatal diagnosis was recommended for 16.7% (82/492), but it was actually performed in only 8.3% (41/492), and the PDR was 50% (41/82). There was no significant difference in the PDR between the NIPT high-risk and low-risk groups.

**Conclusions:**

The screening performance of NIPT for SCA in twin pregnancies needs to be further evaluated. When abnormal NIPT results or USG findings are used as the main prenatal diagnostic indicator in the second and third trimesters, the PDR is poor.

## Background

Since its clinical application in 2011, noninvasive prenatal testing (NIPT) has shown high sensitivity and specificity in screening for trisomy (T) 21, 18, and 13 in singleton pregnancies [1]. In twin pregnancies, the detection rate and false-positive rate of NIPT for T21 were 99.0% and 0.02%, respectively [2], which were superior to those of first-trimester combined tests or second-trimester biochemical tests [3, 4]. According to current studies, it is not possible to evaluate the screening performance of NIPT for T18 and T13 because of the small number of positive cases [5]. In large studies of singleton pregnancies, the incidence of sex chromosome aneuploidy (SCA) is second only to those of T21 and T18 in prenatal screening [6], and SCA may lead to developmental disorders of secondary sexual characteristics, infertility, motor and language deficits, mental retardation and behavioural problems [7]. Therefore, it is necessary to evaluate the screening performance of NIPT for SCA in twin pregnancies, but there are few clinical studies on this aspect. A study reported on seven twin pregnancies with NIPT results suspected to indicate SCA, all of which were confirmed as false-positives by karyotyping [8]. Therefore, more clinical studies are needed to validate the feasibility of NIPT for screening common chromosomal aneuploidies and SCA in twin pregnancies.

In the clinical prenatal examination of twin pregnancy, ultrasound screening is equally important as aneuploidy screening. Ultrasound can determine chorionicity, screen for foetal structural malformations and serve as a complementary means to reveal whether a foetus suffers from a chromosomal abnormality. Invasive prenatal diagnosis is recommended when the NIPT result indicates a high risk of abnormalities or when ultrasound suggests a foetal structural malformation. Amniocentesis in twin pregnancies carries a slightly higher risk of foetal loss than in singleton pregnancies [9], and many twin pregnancies are conceived through artificially assisted reproduction or at an advanced age; therefore, it is a common phenomenon for twin-pregnant women to refuse prenatal diagnosis after a long period of hesitation.

In this study, we aimed to retrospectively analyse the screening performance of NIPT for foetal chromosomal abnormalities and the prenatal diagnosis rate (PDR) when abnormal NIPT results or ultrasonographic (USG) findings are the main prenatal diagnostic indicator in twin pregnancies.

## Methods

### Participants

The NIPT inclusion criterion for this study was twin pregnancies at ≥ 12 weeks of gestation. The NIPT exclusion criteria for this study were as follows: (1) pregnant women for whom ultrasound indicated nuchal translucency thickness ≥ 3.0 mm or a structural malformation in at least one of the twins before NIPT; (2) couples in which one or both partners suffered from definite chromosomal abnormalities or a family history of genetic diseases; (3) pregnant women who suffered from malignant tumour or had undergone transplantation, stem cell therapy, or allogeneic blood transfusion within 1 year; and (4) pregnant women with vanishing twin syndrome for whom the time of blood collection was less than 8 weeks after the death of one foetus. Women with twin pregnancies who met the aforementioned criteria and underwent NIPT at the prenatal diagnostic centre of Xiangya Hospital, Central South University from January 2018 to May 2022 were included in the study. Pregnant women were informed of the content and limitations of the test and signed an informed consent form prior to the test.

### NIPT procedure

Maternal peripheral blood (8–10 ml) was collected, and the plasma was separated within 8 h with EDTA anticoagulant tubes or within 72 h with cell-free DNA (cfDNA) BCT tubes. cfDNA extraction and library construction were performed according to the JingXin Fetal Chromosome Aneuploidy (T21, T18, and T13) Testing Kits (Boao Bio-Tech Co., Ltd., Beijing, China). Semiconductor sequencing was performed on the BioelectronSeq 4000 Platform (Thermo Fisher, Waltham, MA, USA). The threshold for the foetal fraction was set at 4%. After bioinformatics analysis, Z scores were used to assess foetal chromosome aneuploidy and microdeletion/microduplication.

### Ultrasound screening and grouping

An ultrasound scan was carried out between 11 and 13^+ 6^ gestational weeks to determine chorionicity, measure nuchal translucency thickness and screen for foetal malformations. According to ISUOG practice guidelines, routine twin ultrasound monitoring was performed every two to four weeks depending on chorionicity, and more frequent ultrasound scans were performed in complicated pregnancies [10]. Except for those with spontaneous miscarriage or termination of pregnancy, each twin-pregnant woman was followed up by ultrasound for at least 28 gestational weeks. USG findings included ultrasonographic soft markers (USMs), structural malformations (SMs), foetal growth restriction (FGR) and anatomical variations, which could occur in either or both twins. The diagnostic criteria for FGR in twin pregnancies were established according to expert consensus [11]. Pregnancies with complications related to monochorionic twins, such as twin-to-twin transfusion syndrome and conjoined twins, were excluded.

With the combination of NIPT results and USG findings, twin pregnancies were divided into the following groups: (1) Group A: twin pregnancies with NIPT results indicating a high risk of abnormalities; (2) Group B: twin pregnancies with NIPT results indicating a low risk of abnormalities and USG findings for one of the twins; GB-1 (Group B-1): one of the twins had USMs; GB-2: one of the twins had a SM; GB-3: one of the twins had USMs and a SM; GB-4: one of the twins had FGR; and GB-5: one of the twins had other abnormalities; and (3) Group C: twin pregnancies with NIPT results indicating a low risk of abnormalities and USG findings for both twins; GC-1 (Group C-1): at least one of the twins had USMs; GC-2: at least one of the twins had a SM; and GC-3: at least one of the twins had FGR or other abnormalities.

### Genetic counselling and prenatal diagnosis

**Fig. 1:**
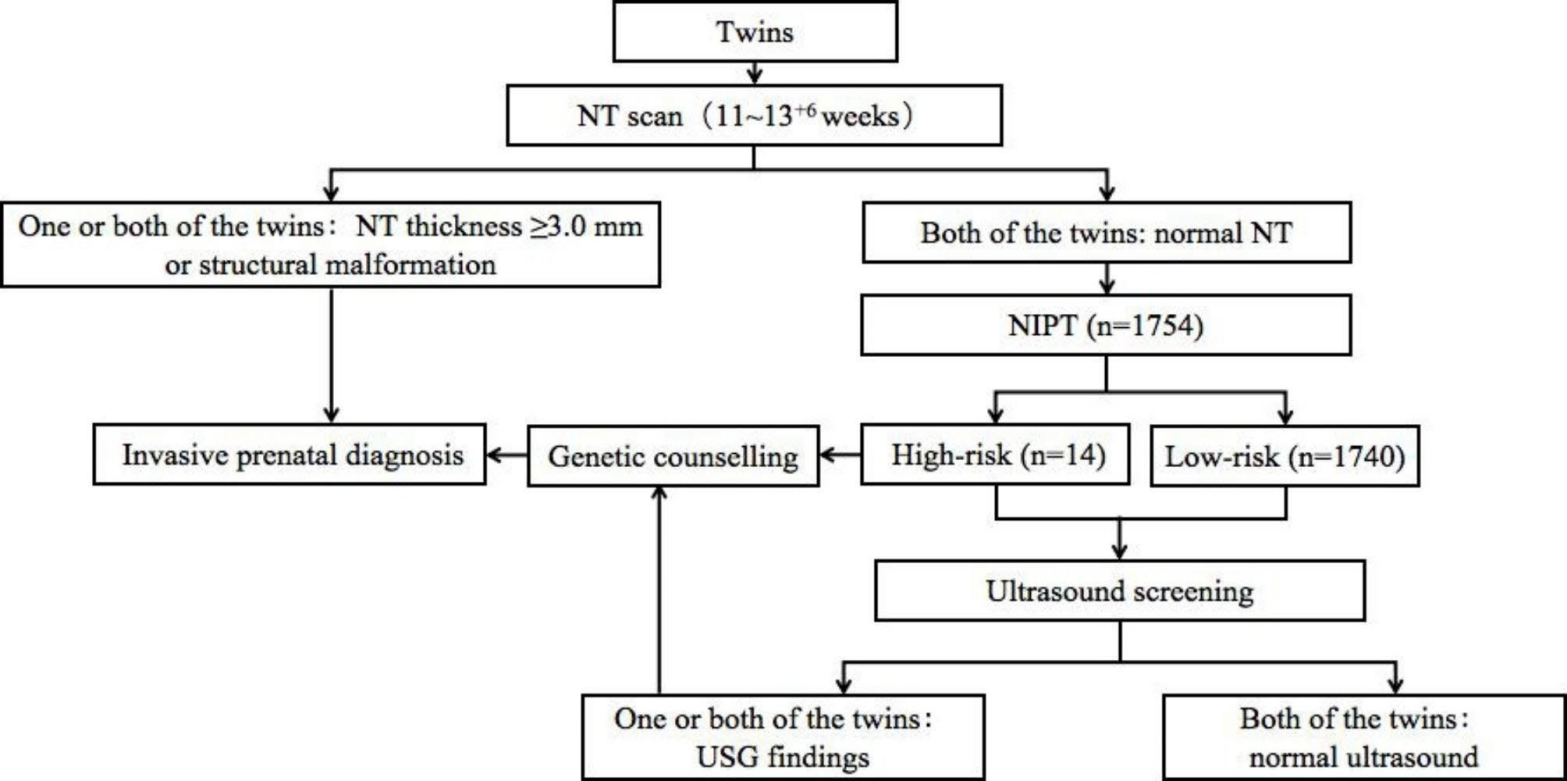
The brief flow chart for screening with ultrasound and NIPT in twin pregnancy NIPT: noninvasive prenatal testing; NT: nuchal translucency; USG: ultrasonographic

If the NIPT result indicated high risk or ultrasound indicated a SM in at least one of the twins, prenatal diagnosis was recommended. If the NIPT result indicated low risk and USG findings indicated nonstructural malformations (such as an USM or anatomical structure variation), comprehensive evaluation was conducted in combination with maternal age or serological screening. Prenatal diagnosis was recommended if there were two or more USMs, the maternal age was older than 35 years, or the serological screening result indicated high risk. Amniocentesis was the preferred sampling method for prenatal diagnosis, and umbilical cord blood puncture was selected for failure of amniocentesis. Karyotyping was performed by the conventional Giemsa banding method, and chromosomal microarray analysis was processed using a CytoScan 750 K array (Affymetrix, Santa Clara, CA, USA) according to the manufacturer’s instructions. A brief flow chart for screening with ultrasound and NIPT is shown in Fig. 1.

### Follow-up

The follow-up contents included the pregnancy outcomes of twin pregnancies and the health status of newborns. All newborns were examined by paediatricians, and those suspected of chromosomal abnormalities were further examined and diagnosed. The prenatal diagnostic centre and communities followed up the newborns to track their growth and development.

### Statistical analysis

SPSS version 26 software was used for statistical analysis. Numerical variables are expressed as the mean ± standard deviation, and categorical variables are expressed as percentages. Screening performance was evaluated using the screening test method, such as sensitivity [true positives/(true positives + false negatives)], specificity [true negatives/(true negatives + false positives)] and positive predictive value (PPV) [true positives/(true positives + false positives)]. The PDR was defined as the number of actual prenatal diagnoses performed divided by the number of recommended prenatal diagnoses. The chi-square test was used to compare the PDR between different groups, and a P value < 0.05 was considered statistically significant.

## Results

### Screening performance of NIPT for foetal chromosomal abnormalities in twin pregnancies

NIPT was successfully performed for 1754 twin pregnancies in the prenatal diagnostic centre. The age of the pregnant women ranged from 17 ~ 53 years (30.5 ± 4.3). The gestational weeks of blood sampling ranged from 12 ~ 25 weeks (16.9 ± 2.0). There were 702 (40.0%) cases of natural pregnancy and 1052 (60.0%) cases of artificially assisted reproduction. A total of 14 twin pregnancies with NIPT results indicating high risk were detected, and their clinical and laboratory data are summarized in Table 1. The NIPT results of three twin pregnancies (Cases 12–14) who refused prenatal diagnosis were as follows: one case of SCA, one case of chromosome 15q21.3-q23 deletion (10 Mb) and one case of T7. No false-negative cases were reported in our study. The screening performance of NIPT for foetal chromosomal abnormalities in twin pregnancies is summarized in Table 2. The sensitivity, specificity and PPV of NIPT for T21 were 100%, 99.9% and 75%, and the corresponding values for SCA were 100%, 99.9% and 50%, respectively. One case of T18 and two cases of microdeletion/microduplication syndrome (MMS) detected by NIPT were all false-positives. In terms of USG findings, Case 1 and Case 5 were vanishing twins. In Case 3, the Twin B foetus was diagnosed with polyhydramnios at 27^+ 6^ gestational weeks and FGR at 32^+ 2^ gestational weeks. In Case 11, the Twin B foetus was diagnosed with an aberrant right subclavian artery at 22^+ 3^ gestation weeks. No ultrasound abnormalities were found in the other pregnancies.

**Table 1:** The clinical and laboratory data of 14 twin pregnancies with NIPT results indicating high risk

Case no.	MA	GW	Chorionicity	NIPT Results	USG Findings	PD Method	PD Results	Outcomes
1	33	20^+ 5^	DCDA	T21	TwinA: embryo arrest at 9 GW TwinB:N	AC	TwinB:N	TwinB:LB
2	38	16^+ 2^	MCDA	T21	TwinA and TwinB:N	AC	TwinA and TwinB:T21	TwinA and TwinB:TOP
3	33	19^+ 3^	DCDA	T21	TwinA:N TwinB:FGR, PH	AC	TwinA:N TwinB:T21	TwinA:LB TwinB:FR
4	36	15^+ 3^	DCDA	T21	TwinA and TwinB:N	AC	TwinA:T21 TwinB:N	TwinA:FR TwinB:LB
5	24	23^+ 3^	DCDA	T18	TwinA:embryo arrest at 12 GW TwinB:N	AC	TwinB:N	TwinB:LB
6	28	15^+ 5^	DCDA	XXY	TwinA and TwinB:N	AC	TwinA:N TwinB:47,XXY	TwinA:LB^a^ TwinB:FR
7	29	16	DCDA	XXY	TwinA and TwinB:N	AC	TwinA:47,XXY TwinB:N	TwinA and TwinB:SA
8	28	19^+ 1^	DCDA	XXX	TwinA and TwinB:N	AC	TwinA and TwinB:N	TwinA and TwinB:LB
9	33	16^+ 1^	DCDA	XO	TwinA and TwinB:N	AC	TwinA and TwinB:N	TwinA and TwinB:LB
10	25	18^+ 4^	DCDA	11q14.3-q25 deletion (45 Mb)	TwinA and TwinB:N	AC	TwinA and TwinB:N	TwinA and TwinB:LB
11	33	16^+ 6^	DCDA	DiGeorge syndrome	TwinA:N TwinB:ARSA	AC	TwinA and TwinB:N	TwinA and TwinB:LB
12	23	16^+ 1^	DCDA	XXX	TwinA and TwinB:N	-	-	TwinA and TwinB:LB
13	34	16^+ 2^	DCDA	15q21.3-q23 deletion (10 Mb)	TwinA and TwinB:N	-	-	TwinA and TwinB:LB
14	28	16	MCDA	T7	TwinA and TwinB:N	-	-	TwinA and TwinB:LB

AC:amniocentesis; ARSA:aberrant right subclavian artery; DCDA: dichorionic diamnion; FGR:fetal growth restriction; FR:fetal reduction; GW:gestational weeks; LB: live birth; MA: maternal age; MCDA: monochorionic diamnion; N: normal; PD: prenatal diagnosis; PH: polyhydramnios; SA: spontaneous abortion; T: trisomy; TOP: termination of pregnancy; USG: ultrasonographic

^a^:The infant was diagnosed with congenital carnitine deficiency at age two

**Table 2:** The screening performance of NIPT for foetal chromosomal abnormalities in twin pregnancies

chromosomal abnormalities	Detected	True positive	False positive	True Negative	Sensitivity (95% CI)	Specificity (95% CI)	PPV (95% CI)
T21	4	3	1	1750	100%(29.2–100)	99.9%(99.7–99.9)	75%(19.4–99.4)
T18	1	0	1	1753	-	99.9%(99.7–99.9)	-
SCA	5	2	2	1749	100%(15.8–100)	99.9%(99.6–99.9)	50%(6.8–93.2)
MMS	3	0	2	1751	-	99.9%(99.6–99.9)	-

CI: confidence intervals; MMS: microdeletion/microduplication syndrome; PPV: positive predictive value

### USG findings and prenatal diagnosis of twin pregnancies with NIPT results indicating low risk

Our hospital is a regional referral centre with an integral prenatal screening and diagnosis system. Many twin-pregnant women who underwent NIPT did not receive routine prenatal examinations in our hospital, so we were only able to track the ultrasound manifestations, prenatal diagnosis results and pregnancy outcomes of 492 twin pregnancies with NIPT results indicating low risk. The clinical data of the 492 twin pregnancies are shown in Table 3. Follow-up results of foetal ultrasound manifestations showed that there were 144 pregnancies with USG findings for one of the twins, including 81 cases of USMs, 11 cases of SMs, 7 cases of USMs and SMs, 29 cases of FGR, and 16 cases of other abnormalities. In addition, 50 pregnancies had USG findings for both twins, including 20 cases of at least one twin with USMs, 8 cases of at least one twin with SMs, and 22 cases of at least one twin with FGR or other abnormalities. Among the USG findings for one twin, the top three were choroid plexus cyst, FGR and pyelectasis, and the most common structural malformation was ventricular septal defect. After genetic counselling, prenatal diagnosis was recommended for 52 pregnancies, and prenatal diagnosis was performed for only 26. Among USG findings for both twins, the top two were FGR and choroid plexus cyst. After genetic counselling, prenatal diagnosis was recommended for 30 pregnancies, and prenatal diagnosis was performed for only 15. USG findings and prenatal diagnosis details are shown in Table 4. Our study showed that if both twins had nuchal translucency thickness ˂3.0 mm and no structural malformations in the first trimester, the rate of USG findings in twin pregnancies in the second and third trimesters with NIPT results indicating low risk was 39.4% (194/492); of these pregnancies, prenatal diagnosis was recommended for 16.7% (82/492), but prenatal diagnosis was performed for only 8.3% (41/492), and the PDR was 50% (41/82).

**Table 3:** The clinical data of 492 twin pregnancies with NIPT results indicating low risk

Characteristic	Cases (n, %)
**Age**	
23–35	398(80.9%)
35–53	94(19.1%)
**Chorionicity**	
DCDA	425(86.4%)
MCDA	65(13.2%)
MCMA	2(0.4%)
**Types of pregnancy**	
artificially assisted reproduction	418(85.0%)
natural pregnancy	74(15.0%)
**Pregnancy outcomes**	
twin live births	452(91.9%)
embryo arrest/live birth	6(1.2%)
stillborn/live birth	7(1.4%)
intrauterine fetal reduction/live birth	3(0.6%)
twin termination of pregnancy	1(0.2%)
twin spontaneous abortion	9(1.8%)
twin pregnancies on-going	14(2.9%)
**Ultrasound manifestations**	
USG findings in one of the twins	144(29.3%)
USG findings in both of the twins	50(10.1%)
Normal ultrasound in both of the twins	298(60.6%)

DCDA: dichorionic diamnion; MCDA: monochorionic diamnion; MCMA: monochorionic monoamnion; USG: ultrasonographic

**Table 4:** The USG findings and prenatal diagnosis of 492 twin pregnancies with NIPT results indicating low risk

GroupB-1		GroupB − 2		GroupB-3		GroupB-4		GroupB-5			GroupC-1	GroupC-2		GroupC-3			
Type	No.^a^	Type	No.	Type	No.	Type	No.		Type	No.	Type(A/B)	No.	Type(A/B)	No.	Type(A/B)	No.	
CPC	36	MM	2^††^	VSD + CPC	3^†††^	FGR	19^†††‡‡‡‡^		PH	9^†^	CPC/CPC	6	SD/CPC	1^‡^	FGR/FGR	9^††‡‡‡‡‡^	
PE	19	VSD	3^†‡‡^	VSD + AC + NT	1^†^	FGR + CPC	3		SB	1^‡^	PE/PE	2	PEV + CD/CD	1^‡^	FGR + CPC/CPC	1	
VM	8^†‡‡‡‡^	VR	1^†^	AC + VM	1^‡^	FGR + VM	1^‡^		SB + SUA	1^‡^	CPC/SUA	2	BM/FGR	1^†^	FGR/CPC	1^‡^	
ARSA	7^††‡‡^	SPD	1^†^	AC + PLSVC + PE	1^†^	FGR + NT	1^†^		LC	2	CPC/CPC + PE	1	AOLK/PEV	1^†^	FGR + HI/ECM	1	
NT	3^‡‡‡^	EK	1^‡^	NT + PEV	1^†^	FGR + SUA	1		ARA	1	CPC/PE	1	VSD/CPC + FGR	1^†^	FGR + VM/ARSA	1^†^	
SUA	3	PPD	1^‡^			FGR + SCD	1^†^		NDC	1	CPC/DIVC	1	CD/SUA + FGR	1^‡^	FGR/SUA + PH	1^†^	
HI	2	TOF	1^†^			FGR + PH	1^‡^		CC	1^‡^	CPC/VM	1^‡^	DA/HI	1^‡^	FGR/PRUV	1^‡^	
NBA	1^‡^	MM + SB	1^†^			FGR + HI + SB	1^‡^				ARSA/SB	1^‡^	PK/CPC	1^†^	FGR/SEC	1^†^	
PRUV	1					FGR + VM + SB	1^†^				EIF/EIF	1			FGR/FGR + PH	1^†^	
PLSVC	1										HI/HI	1^‡^			FGR + CPC/FGR	2^†‡^	
RAA	1^†^										NBA/NBA	1^†^			FGR/FGR + SB	1^†^	
GD	1^†^										NT/PE	1^†^			CNPT/CNPT	1	
ECM	1										VM/VM	1^†^			GE/NDC	1	
WCV	1^‡^																
AOVC	1^†^																
SFL	1																
Total	81		11		7		29			16		20		8		22	
RPD	17		11		7		13			4		6		8		16	
APD	6		7		6		6			1		3		4		8	
PDR	35.3%		63.6%		85.7%		46.2%			25.0%		50.0%		50.0%		50.0%	
Results	1VUS		3VUS		N		1VUS			N		N		N		2VUS	

Group B: twin pregnancies with NIPT results indicating a low risk of abnormalities and USG findings for one of the twins; GB-1 (Group B-1): one of the twins had USMs; GB-2: one of the twins had a SM; GB-3: one of the twins had USMs and a SM; GB-4: one of the twins had FGR; GB-5: one of the twins had other abnormalities; Group C: twin pregnancies with NIPT results indicating a low risk of abnormalities and USG findings for both twins; GC-1 (Group C-1): at least one of the twins had USMs; GC-2: at least one of the twins had a SM; GC-3: at least one of the twins had FGR or other abnormalities

AC: aortic coarctation; AOLK: absence of left kidney; AOVC: absence of venous catheters; ARA: accessory renal artery; ARSA: aberrant right subclavian artery; BM: brain malformation;

CC: choledochal cyst; CD: cerebellum dysplasia; CNPT: cystic nodules posterior thalamus; CPC: choroid plexus cyst; DA: duodenal atresia; DIVC: double inferior vena cava; ECM: enlarged cisterna magna; EIF: echogenic intracardiac focus; EK: ectopic kidney; GD: gallbladder dysplasia; GE: gallbladder enlargement; HI: hyperechogenic intestine; LC: liver cyst; MM: multiple malformations; N: normal; NBA: nasal bone aplasia; NDC: nasolacrimal duct cyst; NT: increased nuchal translucency(95th˂NT˂3.0 mm); PE: pyelectasis; PEV: pes equinovarus; PH: polyhydramnios; PK: polycystic kidney; PLSVC: persistent left superior vena cava; PPD: preaxial polydactyly; PRUV: persistent right umbilical vein; RAA: right aortic arch; SB: stillbirth; SCD: scaphocephaly deformity; SD: spinal deformity; SEC: subependymal cyst; SFL: short femur length; SM: structural malformation; SPD: septum pellucidum dysplasia; SUA: single umbilical artery; TOF: tetralogy of fallot; USG: ultrasonographic; USM: ultrasonographic soft marker; VM: ventriculomegaly; VR: ventricular rhabdomyoma; VSD: ventricular septal defect; WCV: widened cavum vergae

RPD: recommended prenatal diagnosis; APD: accepted prenatal diagnosis; PDR: prenatal diagnosis rate;VUS: variants of uncertain significance

^a^: Two soft markers were found in the same fetus (6 cases): CPC + HI、CPC + PE(2 cases)、CPC + VM、CPC + NT、ARSA + PE

^†^: prenatal diagnosis was recommended and performed

^‡^: prenatal diagnosis was recommended and not performed

### Comparison of the PDR between twin pregnancies with NIPT results indicating high and low risk

Eleven out of the 14 twin pregnancies with NIPT results indicating high risk (Group A) underwent prenatal diagnosis, and the PDR was 78.6% (11/14). Among the twin pregnancies with NIPT results indicating low risk and USG findings for one of the twins (Group B), the PDR ranged from 25.0 to 85.7% according to different ultrasound phenotypes. The PDR was the highest when the phenotype was USMs combined with SMs. The total PDR was 50.0% (26/52). Among the twin pregnancies with NIPT results indicating low risk and USG findings for both twins (Group C), the PDR was 50% in all groups. In conclusion, the total PDR was 50% in twin pregnancies with NIPT results indicating low risk. There was no significant difference in the total PDR among Groups A, B and C. The acceptance rate of NIPT in our study population was 100%, but the total PDR did not exceed 80% when abnormal NIPT results or USG findings were used as an indication for prenatal diagnosis, which revealed a poor PDR in the second and third trimesters of twin pregnancies.

In Group A, all three twin-pregnant women who refused prenatal diagnosis had live births. In Group B, five twin-pregnant women with foetuses with SMs did not receive prenatal diagnosis. The reasons and pregnancy outcomes were as follows: prenatal diagnosis was refused for two cases of ventricular septal defect and one case of preaxial polydactyly, and all the twins were born alive; prenatal diagnosis was contraindicated for one case of ectopic kidney with acute heart failure and pulmonary infection, and the twins were born alive after treatment; and one foetus with aortic coarctation + ventriculomegaly was diagnosed by ultrasound at 33^+ 4^ gestational weeks and delivered after premature rupture of membranes at 35^+ 4^ gestational weeks. Of the remaining 21 twin pregnancies without prenatal diagnosis, in 19 pregnancies, the twins were born alive, and in two pregnancies, they were stillborn/born alive. In Group C, prenatal diagnosis was not performed for four twin pregnancies with SMs. The reasons and pregnancy outcomes were as follows: for the twin pregnancy with spinal deformity/choroid plexus cyst, the foetus with spinal deformity was reduced, while prenatal diagnosis was refused for the surviving foetus, who was born alive; prenatal diagnosis was refused for the twin pregnancy with pes equinovarus + cerebellum dysplasia/cerebellum dysplasia and the pregnancy was terminated; prenatal diagnosis was refused for the twin pregnancy with cerebellum dysplasia/single umbilical artery + FGR and the twins were born alive; and the twin pregnancy with duodenal atresia/hyperechogenic intestine was not suitable for prenatal diagnosis due to urinary calculi combined with sepsis and cervical insufficiency, and twins were born alive after treatment. Of the remaining 11 twin pregnancies without prenatal diagnosis, in 10 pregnancies, the twins were born alive, and in one pregnancy, the twins were stillborn/born alive. The reasons for not performing prenatal diagnosis in 44 cases of twin pregnancies were summarized as follows: (1) 70.5% (31/44) of pregnant women refused prenatal diagnosis for fear of foetal loss; (2) 4.5% (2/44) of severely malformed foetuses were directly reduced or terminated; (3) 15.9% (7/44) of pregnant women had contraindications for prenatal diagnosis, such as threatened abortion, cervical insufficiency or infection; and (4) 9.1% (4/44) of pregnant women were at high gestational age when ultrasound abnormalities were detected.

## Discussion

In this study, we found that NIPT has good screening performance for T21 and SCA in twin pregnancies. The sensitivity and specificity of NIPT for T21 were 100% and 99.9%, respectively, which are consistent with the results of singleton or other twin studies [12–14]. For SCA, NIPT has a different sensitivity and specificity according to the types of SCA in singleton pregnancies, and the PPV is between 21.4% and 90.9% [15, 16]. In our study, the sensitivity and specificity of NIPT for SCA were 100% and 99.9%, respectively, but the PPV was lower than that of T21. The ultrasound phenotypes of SCA are often mild and sometimes even normal on prenatal screening, so due to the high prevalence of SCA but the lack of better screening methods at present, we prefer to perform NIPT screening for SCA in twin pregnancies. However, because the number of SCAs detected in our study was small, more clinical data need to be accumulated to further evaluate the screening performance of NIPT for SCA in twin pregnancies. The PPV of NIPT for MMS in singleton pregnancies ranges from 28.99–49.02% [17, 18], and two cases of MMS detected by NIPT in our study were both false-positives. No true-positive cases of T18, T13 and MMS were detected in our study, so the screening performance of NIPT for these abnormalities could not be assessed. As NIPT becomes more widely used in twin pregnancies, more data will likely emerge to analyse the screening performance of NIPT for T18, T13, and MMS.

The study revealed that the PDR is poor in twin pregnancies when abnormal NIPT results or USG findings are used as an indication for prenatal diagnosis. When the NIPT results indicated high risk, the PDR was 78.6%, and those who refused prenatal diagnosis were suspected of chromosomal abnormalities other than common aneuploidies. When the NIPT results indicated low risk, the total PDR of twin pregnancies with USG findings was 50%. The possible reasons for not performing prenatal diagnosis include the following: (1) fear of foetal loss due to the invasiveness of prenatal diagnosis; (2) direct reduction or termination of pregnancy for severely malformed foetuses; (3) contraindications for prenatal diagnosis; and (4) the gestational age at which the ultrasound abnormalities were detected was too high, and prenatal diagnosis results may not be available before delivery. The poor PDR resulted in a proportion of twin pregnancies not receiving the genetic diagnosis, increasing the risk of foetal birth defects. A retrospective study has shown that the diagnostic yield of pathogenic copy number variation in prenatal cases is lower than that in postnatal cases [19]. Therefore, we believe that for twin pregnancies with prenatal diagnostic indications, improving the PDR and extending prenatal diagnosis to more prenatal cases can improve the diagnostic yield of cytogenomic abnormalities. Hower, how to improve the PDR is a complex problem involving many factors. Clinically, the improvement of prenatal diagnostic sampling techniques, standardized ultrasound screening, appropriate pregnancy management, and good genetic counselling are helpful to improve the PDR of twin pregnancy. The current study showed that the risk of foetal loss following amniocentesis or chorionic villus sampling in twins did not differ from the background risk in twin pregnancies for which prenatal diagnosis was not performed [20]. Therefore, genetic counselling should not only provide information on the risk of invasive prenatal diagnosis but also alleviate the anxiety of pregnant women who are worried about foetal loss, especially for those with assisted reproductive conception or advanced age.

USG findings account for a significant proportion of twin pregnancies with NIPT results indicating low risk. The current routine prenatal genetic testing for twin pregnancies, including karyotyping and chromosomal microarray analysis, may not meet the clinical testing needs. For twin pregnancies in which both twins had nuchal translucency thickness ˂3.0 mm and no foetal structural malformations in the first trimester, USG findings were detected in 39.4% of the twin pregnancies with NIPT results indicating low risk in the second and third trimester, including USMs, SMs, FGR, and anatomic variations. SMs in one or both twins accounted for 5.3% of the pregnancies. However, no aneuploidies or pathogenic copy number variations were found in the prenatal diagnosis results. A low PDR and limited genetic testing techniques may lead to some twin-pregnant women with abnormal ultrasound phenotypes not receiving or obtaining false-negative genetic diagnoses. Karyotyping and chromosomal microarray analysis may miss the diagnosis of single-gene disorders (Case 6), which is limited by the molecular diagnostic level of genetics. It has been reported that for foetal structural abnormalities with normal karyotype and chromosomal microarray analysis results, whole-exome sequencing can improve the genetic diagnosis rate by 8.5-10.0% [21, 22]. With the accumulation of intrauterine ultrasound phenotype-genotype databases, whole-exome sequencing will be gradually applied in the prenatal diagnosis of twin pregnancies.

The disadvantage of this study is that the number of twin pregnancies with NIPT results indicating low risk that were lost to follow-up was large. Although the prenatal diagnosis results and pregnancy outcomes of twin pregnancies can be followed up by telephone, it is difficult for most pregnant women to clearly describe the specific ultrasound manifestations during the whole pregnancy, and there are also differences in the level of ultrasound screening in different hospitals. Therefore, only pregnant women who underwent routine prenatal examinations in our hospital were included.

## Conclusions

In summary, our study showed that NIPT has good screening performance for T21, and its screening performance for SCA needs to be further evaluated. For twin pregnancies with nuchal translucency thickness ˂3.0 mm and no foetal structural malformations in the first trimester, when abnormal NIPT results or USG findings are used as the main prenatal diagnostic indicator in the second and third trimesters, the PDR is poor.

## Data Availability

All data generated or analysed during this study are included in this published article.

## Abbreviations

FGR: Foetal growth restriction
MMS: Microdeletion/microduplication syndrome
NIPT: Noninvasive prenatal testing
PDR: Prenatal diagnosis rate
PPV: Positive predictive value
SCA: Sex chromosome aneuploidy
SM: Structural malformation
T: Trisomy
USG: Ultrasonographic
USM: Ultrasonographic soft marker
